# Discontinuation of antidepressant treatment: a retrospective cohort study on more than 20,000 participants

**DOI:** 10.1186/s12991-023-00480-z

**Published:** 2023-11-24

**Authors:** Luis M. Garcia-Marin, Aoibhe Mulcahy, Enda M. Byrne, Sarah E. Medland, Naomi R. Wray, Freddy Chafota, Penelope A. Lind, Nicholas G. Martin, Ian B. Hickie, Miguel E. Rentería, Adrian I. Campos

**Affiliations:** 1 Mental Health & Neuroscience Program, QIMR Berghofer Medical Research Institute, Brisbane, QLD Australia; 2 School of Biomedical Sciences, Faculty of Medicine, The University of Queensland, Brisbane, QLD Australia; 3 School of Biomedical Sciences, Faculty of Health, Queensland University of Technology, Brisbane, QLD Australia; 4 Institute for Molecular Bioscience, The University of Queensland, Brisbane, QLD Australia; 5 Queensland Brain Institute, The University of Queensland, Brisbane, QLD Australia; 6 Child Health Research Centre, The University of Queensland, Brisbane, QLD Australia; 7 Brain and Mind Centre, University of Sydney, Camperdown, NSW Australia

**Keywords:** Antidepressant treatment, Discontinuation, Side effects, Comorbidities, SSRI, SNRI

## Abstract

**Background:**

Factors influencing antidepressant treatment discontinuation are poorly understood. In the present study, we aimed to estimate the prevalence of antidepressant treatment discontinuation and identify demographic characteristics, psychiatric comorbidities, and specific side effects associated with treatment discontinuation.

**Methods:**

We leveraged data from the Australian Genetics of Depression Study (AGDS; *N* = 20,941) to perform a retrospective cohort study on antidepressant treatment discontinuation. Participants were eligible if they were over 18 years of age, had taken antidepressants in the past 4 years, and provided informed consent.

**Results:**

Among the ten antidepressants studied, the highest discontinuation rates were observed for Mirtazapine (57.3%) and Amitriptyline (51.6%). Discontinuation rates were comparable across sexes except for Mirtazapine, for which women were more likely to discontinue. The two most common side effects, *reduced sexual function* and *weight gain,* were not associated with increased odds of treatment discontinuation. *Anxiety*, *agitation*, *suicidal thoughts*, *vomiting,* and *rashes* were associated with higher odds for treatment discontinuation, as were lifetime diagnoses of PTSD, ADHD, and a higher neuroticism score. Educational attainment showed a negative (protective) association with discontinuation across medications.

**Conclusions:**

Our study suggests that not all side effects contribute equally to discontinuation. Common side effects such as *reduced sexual function* and *weight gain* may not necessarily increase the risk of treatment discontinuation. Side effects linked to discontinuation can be divided into two groups, psychopathology related and allergy/intolerance.

**Supplementary Information:**

The online version contains supplementary material available at 10.1186/s12991-023-00480-z.

## Background

Depression is among the most common psychiatric disorders. It has a detrimental impact on the quality of life and functioning of individuals. It is estimated to become the leading global cause of disability by 2030 [1], and 70 to 80% of diagnosed patients will experience relapses and recurrences that often require long-term treatment [2].

Pharmacological interventions, which are commonly used as the first line of treatment for depression, are broadly classified as first- or second-generation antidepressants according to their mechanism of action. Although treatment response rates have been estimated around 50% [3], a significant number of individuals will still experience severe residual symptoms of depression, even when undergoing treatment. A lack of treatment response without appropriate medical guidance can lead to treatment discontinuation, which in turn results in relapse, a diminished quality of life, and symptoms such as insomnia, imbalance, nausea, and sensory disturbances [4].

Treatment discontinuation also occurs due to adverse side effects. In a previous study, we described the prevalence, risk factors, and genetic basis of antidepressant side effects in a cohort of 20,941 Australian adults. We discovered that regardless of the antidepressant taken, 60–75% of participants reported at least one side effect. In particular, we identified reduced sexual function and weight gain as the most frequently reported side effects [5]. These findings were reiterated by various long-term studies in which reduced sexual function and weight gain affected up to 70% of individuals who were prescribed antidepressants [6, 7].

Previous research has primarily focused on discontinuation rates from the larger subset of antidepressant drug classes [8, 9], and several studies have observed the highest discontinuation rates in patients who were taking tricyclic antidepressants (TCAs) [8, 10]. In addition, some evidence suggests males are almost twice more likely to discontinue treatment [11]. Previous literature suggests that specific side effects such as skin rash, attempted suicide, headache, and mania are associated with an increased discontinuation from clinical trials of depression treatment in adolescents [12]. Nonetheless, a study seeking to identify potential risk factors for treatment discontinuation, as opposed to discontinuation from clinical trials, is currently lacking. Therefore, in the present study, we explore participant-reported treatment discontinuation due to adverse side effects for ten commonly prescribed antidepressants using a sample of 20,941 participants. We estimate discontinuation rates for each antidepressant and test for association between discontinuation and specific side effects, demographic variables, and psychiatric comorbidities.

## Methods

### AGDS cohort sample

The Australian Genetics of Depression Study (AGDS) recruited 22,424 Australian participants through two streams: a targeted assisted mailout campaign (14%) and an open media campaign (86%). Potential participants were directed to the AGDS website. Ninety-five percent of participants in the AGDS reported being given a diagnosis of depression by a medical practitioner [13]. Informed consent was gathered prior to data collection through online questionnaires after participants received a complete description of the study. The AGDS inclusion criteria included (i) having been prescribed and taken antidepressants and (ii) providing consent to donate a saliva sample for genotyping. A full and detailed description of AGDS recruitment is reported elsewhere [13]. For this study, participants who did not report having a diagnosis of depression were excluded from the analyses (*N* = 1483), yielding a total sample size of 20,941 participants. This study was approved by the QIMR Berghofer Human and Research Ethics Committee under project number P1218.

### Phenotype ascertainment

From the participants that had been diagnosed with depression, they first confirmed they had taken any of the ten most commonly prescribed antidepressants in Australia, listed as Sertraline, Escitalopram, Venlafaxine, Fluoxetine, Citalopram, Desvenlafaxine, Duloxetine, Mirtazapine, Amitriptyline, and Paroxetine. For each antidepressant taken, participants were asked whether they had experienced side effects and, if so, to select which from a checklist of 23 commonly reported antidepressant side effects.

Participants were further asked explicitly whether they stopped taking a specific medication due to adverse side effects. Data on demographics, clinical history, as well as psychiatric traits (such as neuroticism and extraversion) were also gathered. The full list and details of instruments used for AGDS phenotyping are available at https://bit.ly/3y72lyg.

### Statistical analyses

The present study investigated the relationship between participant-reported antidepressant treatment discontinuation and other variables, including demographic factors, side effects, psychiatric comorbidities, and other factors in the Eysenck Personality Questionnaire neuroticism, extraversion scores, and chronotype measures. We assessed these relationships using four multiple logistic regression models, including demographic factors, side effects, psychiatric comorbidities, and Eysenck Personality Questionnaire. This enabled us to quantify associations with antidepressant treatment discontinuation while adjusting for covariate effects, such as the age of treatment initiation and sex. Briefly, logistic regressions are considered extensions of linear models. Therefore, a logistic regression is used to model a linear relationship between predictor variables and the natural logarithm of the odds of a dependent variable [14] . A predominant advantage of logistic regression models is that they enable the estimation of an odds ratio, which in turn indicates the odds of observing a given outcome (i.e., the dependent variable) given a particular exposure (i.e., the predictor variables).

We estimated odds ratios from the effect sizes on the logistic scale and calculated p-values using Wald tests. For every antidepressant, discontinuation was modeled as a binary variable in which cases included participants who stopped antidepressant treatment due to side effects, and controls included participants who did not stop antidepressant treatment due to side effects. All statistical analyses were performed in Python 3.0 using the following modules: pandas, numpy, statsmodels, scipy, seaborn, and matplotlib. Statistical significance was defined using Bonferroni multiple testing correction for each group of variables was performed based on the total number of variables in each group (i.e., 7 demographic factors, 23 side effects, 18 psychiatric comorbidities, and 9 items of the Eysenck Personality Questionnaire; Additional file 1: Tables S1). Nominal associations refer to those with a *p*-value < 0.05.

## Results

### Demographics

**Fig. 1:**
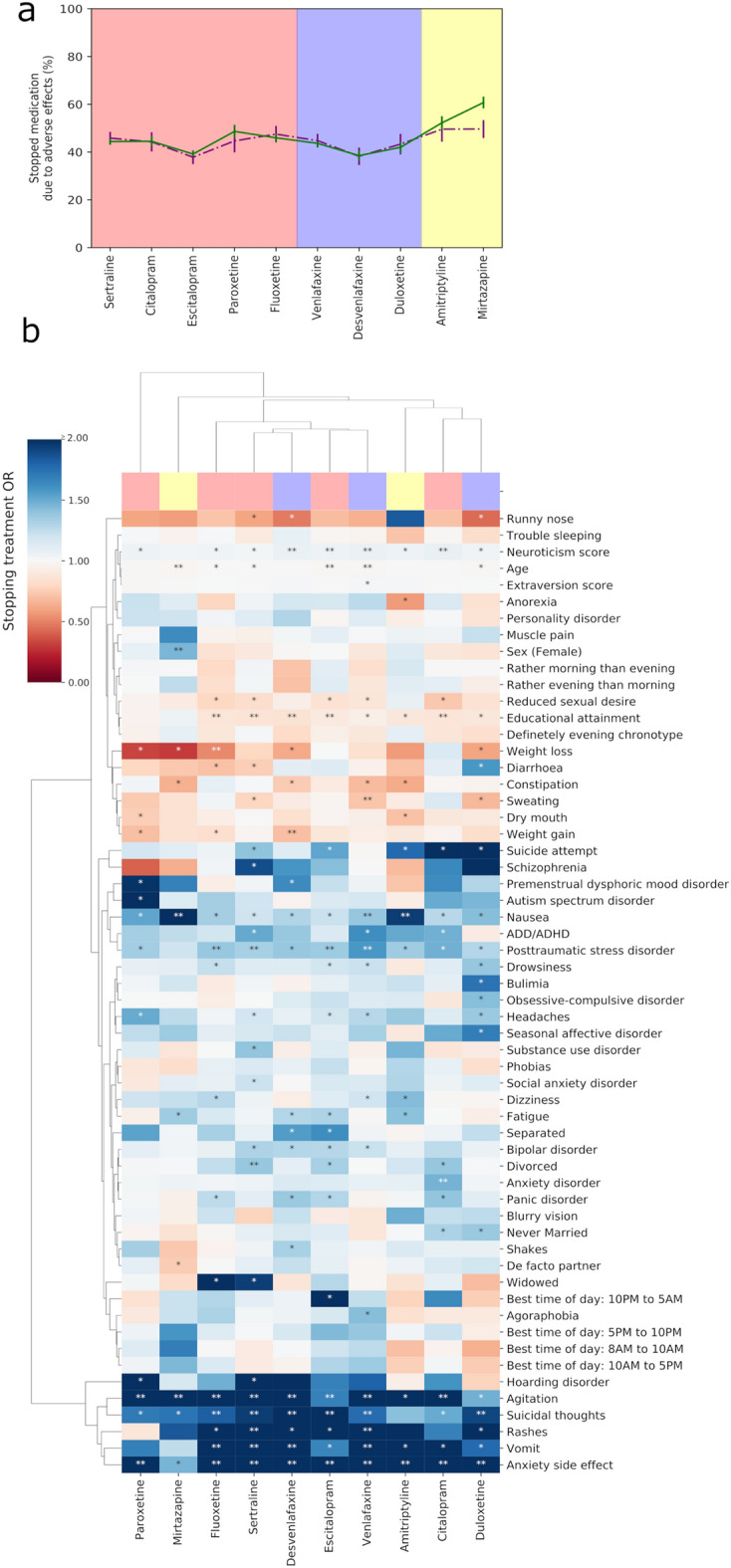
Prevalence and factors associated with treatment discontinuation **a** Estimated discontinuation rates and 95% confidence intervals are shown across medications. The graph is color coded according to medication class (from left to right): SSRIs, SNRIs, and others. Purple dashed lines show the estimates for males, whereas the green solid lines show the results for females. **b** Heatmap depicting the odds ratio between treatment discontinuation and side effects, comorbidities, chronotype, and demographic variables. The color scale represents the odds ratios of associations between these variables and treatment discontinuation. Blue color indicates an increased risk, whereas red color indicates a decreased risk (*protective effect*). *Nominally significant (*p*-value < 0·05) odds ratio. **Significant odds ratio after Bonferroni multiple testing correction.

Table 1 shows the discontinuation rates and key demographics across medications. The present study included a total of 20,941 participants from the AGDS cohort. Of those, 15,830 were women (76%), and 5111 were men (24%). A total of 7723 participants (36·9%) had stopped taking at least one medication due to adverse side effects. We observed the highest discontinuation rate for Mirtazapine (57·3%) and the lowest discontinuation rates for Escitalopram (38.9%) and Desvenlafaxine (38.4%; Fig. 1a).

**Table 1:** AGDS discontinuation prevalence and demographics across medications

Antidepressant	Total discontinuation including both sexes (%)	Controls males (%)	Discontinuation males (%)	Controls females (%)	Discontinuation females (%)	Controls mean age (std)	Discontinuation mean age (std; t; p-value)
Sertraline	2870 (44.7)	738 (54.2)	624 (45.8)	2813 (55.6)	2246 (44.4)	41 (14)	41 (14; 1.65; 0.10)
Escitalopram	1896 (38.9)	674 (62.2)	410 (37.8)	2310 (60.9)	1486 (39.1)	38 (13)	37 (13; − 4.11; 0.00)
Venlafaxine	2017 (43.9)	639 (55.2)	518 (44.8)	1936 (56.4)	1499 (43.6)	43 (14)	43 (14; − 0.57; 0.57)
Amitriptyline	814 (51.6)	176 (50.4)	173 (49.6)	586 (47.8)	641 (52.2)	46 (14)	45 (15; − 0.73; 0.47)
Mirtazapine	1258 (57.3)	338 (50.4)	333 (49.6)	598 (39.3)	925 (60.7)	44 (14)	41 (14; − 4.05; 0.00)
Desvenlafaxine	1078 (38.4)	417 (61.8)	258 (38.2)	1310 (61.5)	820 (38.5)	39 (13)	38 (13; − 2.39; 0.02)
Citalopram	1139 (44.4)	325 (55.7)	258 (44.3)	1099 (55.5)	881 (44.5)	43 (13)	41 (13; − 3.01; 0.00)
Fluoxetine	1774 (46.3)	418 (52.5)	378 (47.5)	1643 (54.1)	1396 (45.9)	40 (15)	41 (15; 0.98; 0.33)
Duloxetine	971 (42.3)	292 (56.7)	223 (43.3)	1034 (58.0)	748 (42.0)	41 (14)	40 (14; − 2.67; 0.01)
Paroxetine	794 (47.7)	228 (55.3)	184 (44.7)	644 (51.4)	610 (48.6)	46 (13)	45 (14; − 0.73; 0.46)

Table showing participants that reported treatment discontinuation by age and sex. Age is the mean age of initiation of antidepressant treatment for participants who had discontinued treatment at the time of the questionnaire. For each antidepressant shown, controls included participants who did not discontinue treatment for the specified antidepressant, but may have discontinued others. Percentages for sex-specific columns were estimated considering the number of cases and controls for the specified sex and antidepressant group. The percentages shown in the column “Total discontinuation including both sexes” were calculated using the number of cases and controls regardless of their sex. Some participants may have discontinued more than one antidepressant throughout their treatment history. Therefore, these participants are considered as cases for all the antidepressants they discontinued. *Two-sample t-test statistics comparing the mean age of discontinuation cases and controls

Women showed higher odds of Mirtazapine discontinuation but not for other antidepressants (Fig. 1a). Divorced participants were more likely to discontinue SSRIs, including Sertraline, Escitalopram, and Citalopram. Further, participants who were never married were more likely to discontinue Citalopram and Duloxetine (Fig. 1b). Higher educational attainment was associated with lower discontinuation of SSRIs such as Citalopram, Escitalopram, Fluoxetine, and Sertraline. We observed a similar effect of high educational attainment on the discontinuation of SNRIs (Fig. 1b).

Participants who reported discontinuation were more likely to have taken antidepressants for less than three months. The proportion of participants who discontinued SSRIs and SNRIs due to side effects was similar across the time scales studied. For all antidepressants, at least 50% of all participants who did not report discontinuation due to side effects took that antidepressant for one year or more. Nonetheless, a sizable proportion of participants stopped taking treatment within a year (Table 2).

**Table 2:** Discontinuation due to adverse effects and duration of treatment

	Stopped due to side effects	Time taking medication			Total
		< 3 months %	4–12 months %	1 + years^a^ %	
Sertraline	Yes	1086 (37·8)	831 (29·0)	933 (32·5)	2870
	No	293 (8·3)	780 (22·0)	2462 (69·3)	3551
Citalopram	Yes	422 (37·1)	360 (31·6)	340 (29·9)	1139
	No	125 (8·8)	312 (21·9)	972 (68·3)	1424
Escitalopram	Yes	714 (37·7)	626 (33·0)	543 (28·6)	1896
	No	249 (8·3)	746 (25·0)	1966 (65·9)	2984
Paroxetine	Yes	303 (38·2)	219 (27·6)	258 (32·5)	794
	No	69 (7·9)	192 (22·0)	600 (68·8)	872
Fluoxetine	Yes	681 (38·4)	554 (31·2)	524 (29·5)	1774
	No	203 (9·8)	489 (23·7)	1348 (65·4)	2061
Venlafaxine	Yes	611 (30·3)	616 (30·5)	774 (38·4)	2017
	No	157 (6·1)	399 (15·5)	1996 (77·5)	2575
Desvenlafaxine	Yes	428 (39·7)	341 (31·6)	304 (28·2)	1078
	No	137 (7·9)	341 (19·7)	1242 (71·9)	1727
Duloxetine	Yes	396 (40·8)	296 (30·5)	262 (27·0)	971
	No	116 (8·7)	269 (20·3)	923 (69·6)	1326
Amitriptyline	Yes	354 (43·5)	230 (28·3)	225 (27·6)	814
	No	131 (17·2)	199 (26·1)	426 (55·9)	762
Mirtazapine	Yes	644 (51·2)	352 (28·0)	241 (19·2)	1258
	No	150 (16·0)	240 (25·6)	532 (56·8)	936

^a^For those who also answered No to “stopped taking due to side effects,” participants may still be taking these antidepressants at the time of reporting

### Side effects

We evaluated the influence of 23 participant-reported side effects on antidepressant treatment discontinuation (Additional file 1: Tables S1). Overall, agitation, vomiting, nausea, rashes, and suicidal thoughts were associated with higher discontinuation rates (Fig. 1b). Conversely, sweating was robustly associated with lower odds of Venlafaxine discontinuation, and nominal associations (p-value < 0.05) were observed for Sertraline and Duloxetine discontinuation (Fig. 1b). Weight gain and reduced sexual drive or function, which are the most common side effects in this sample, did not influence discontinuation rates. In addition, rashes were associated with the discontinuation of Venlafaxine or Sertraline. Treatment discontinuation involving other SNRIs showed nominal associations with rashes, as did some SSRIs such as Escitalopram and Fluoxetine (Fig. 1b).

Suicidal thoughts were associated with higher discontinuation rates for all SNRIs studied. For SSRIs, in contrast, only Sertraline, Escitalopram, and Fluoxetine discontinuation were associated with suicidal thoughts after multiple testing correction. Nonetheless, discontinuation for all other SSRIs showed nominally significant associations with suicidal thoughts, as did Mirtazapine. Suicide attempt was nominally associated with higher discontinuation of several SSRIs, namely, Citalopram, Escitalopram, and Sertraline, whereas for SNRIs, only Duloxetine showed a nominal association with suicide attempt (Fig. 1b).

### Comorbidities and psychiatric traits

We evaluated whether psychiatric traits, such as participant-reported diagnoses of psychiatric disorders, neuroticism score, or chronotype measures, were associated with antidepressant treatment discontinuation (Additional file 1: Tables S1). Of the 18 mental health conditions assessed, we found that ADHD was nominally associated with higher odds of SSRIs discontinuation, including Citalopram and Sertraline, and the SNRI Venlafaxine (Fig. 1b). Furthermore, reported Post-Traumatic Stress Disorder (PTSD) was associated with higher odds of discontinuing treatment that included the SSRIs Sertraline, Escitalopram, or Fluoxetine, whereas for SNRIs, a strong association with treatment discontinuation was only observed for Venlafaxine (Fig. 1b). Discontinuation for other SSRIs and SNRIs was nominally associated with PTSD (Fig. 1b).

Bipolar disorder was nominally associated with higher odds of discontinuing SSRIs such as Sertraline and Escitalopram, as well as SNRIs such as Venlafaxine and Desvenlafaxine (Fig. 1b). Similarly, panic disorder showed nominal associations with higher odds of discontinuing SSRIs such as Citalopram, Escitalopram, and Fluoxetine, while for SNRIs, panic disorder was only nominally associated with Desvenlafaxine. Duloxetine discontinuation was nominally associated with bulimia, obsessive–compulsive disorder, and seasonal affective disorder (Fig. 1b). In addition, we observed nominal associations between Paroxetine discontinuation and hoarding disorder, premenstrual dysphoric mood disorder, and autism spectrum disorder. Neuroticism was associated with higher discontinuation rates across all antidepressants except Mirtazapine (Fig. 1b).

## Discussion

The present study sought to provide an overview of the main variables influencing antidepressant treatment response by investigating which antidepressant side effects, comorbidities, and demographic characteristics were associated with the discontinuation of antidepressant treatment. The most common side effects, *weight gain* and *sexual dysfunction,* were not associated with higher discontinuation rates. Although commonly believed to be negative [14], these side effects showed nominal evidence of being protective against discontinuation for some antidepressants. This finding is in contrast to previous studies suggesting that *weight gain* and *sexual dysfunction* are often associated with premature discontinuation [15, 16]. The nominal protective effect of the side effects identified here may indicate that antidepressants are working, which in turn may increase the tolerability for undesired side effects. Weight increases may be induced by an improvement in appetite as symptoms ease. Furthermore, *sexual dysfunction,* particularly in males, may be explained through the known action of antidepressants on testosterone and dopamine regulation [17, 18].

Specific side effects such as *rashes*, *nausea*, *vomiting*, *anxiety*, *agitation,* and *suicidality* were associated with an increased risk for treatment discontinuation. Our findings are partially consistent with previous observations, which reported that *suicidality*, *mania*, *rashes,* and *headaches* were associated with discontinuation from clinical trials [12]. These results suggest at least two pathways to discontinuation: an intolerance or allergic component manifested as nausea, vomiting, and rashes, and a psychopathology-related component including anxiety and suicidality. Currently, it is challenging to establish whether the latter is explained by a lack of antidepressant effectiveness as opposed to undesired adverse effects.

Studies reporting the effect of divorce on mental health suggest that married individuals are more likely to respond positively to antidepressant treatment [19]. In addition, we found that individuals with high educational attainment are less likely to discontinue antidepressant treatment, which in turn supports evidence from previous genetic studies [20]. Matching our results, discontinuation is recognized as a concern for individuals diagnosed with ADHD and PTSD [21, 22]. For instance, previous studies report low antidepressant effectiveness among patients with comorbid ADHD [23] or PTSD [24]. It is important to distinguish that these associations are more challenging to interpret due to a lack of information on age at onset. This limitation is less likely to affect associations with ADHD as it is typically an early-onset condition; however, we cannot rule out the possibility of participants starting and stopping an antidepressant regime before developing PTSD or ADHD. In addition, we note that the spectral nature of psychiatric disorders [25, 26] may influence our results. For instance, a higher risk for discontinuation among individuals with ADHD and / or PTSD may be indicative of another psychiatric comorbidity, such as personality disorder. Future studies should aim to delineate further effects and interactions between specific psychiatric disorders and treatment discontinuation.

Some limitations need to be acknowledged. We note that the generalizability of our results must be addressed with caution until confirmed in samples from other populations and ethnicities. Retrospective participant-derived reports of side effects and discontinuation may be subject to recall bias and subjective interpretations of participants. Related to this is the lack of information on comorbidity age of onset. For example, although the association between anxiety as a side effect and discontinuation was stronger compared to anxiety as a previous diagnosis, we do not have sufficient data to assess whether participants who reported anxiety as a side effect had a previous diagnosis of anxiety disorder which could continue through and after treatment. Similarly, due to the nature of the available data, we are not able to know the magnitude of certain side effects such as weight gain. For instance, it may be possible that participants in AGDS experienced weight gain that was insignificant to them and decided to continue their treatment, which is subject to personal interpretations and tolerability. The relationship between discontinuation due to adverse side effects and due to lack of efficacy is complex since these constructs are not independent. A patient could experience severe side effects but high efficacy, potentially increasing the patient’s tolerability to the side effects. However, if a patient experiences low efficacy, it is possible that the patient’s tolerability to side effects will be much lower, increasing the odds of discontinuation. Future studies should aim to further explore the intricate association between tolerability, discontinuation due to adverse side effects, and discontinuation due to lack of efficacy, which was out of the scope of this study.

We note that patients who discontinued more than one drug were considered in analyses for all the drugs they discontinued. Therefore, the effect of individual vulnerabilities predisposing participants to discontinue treatment may be exacerbated. To prevent this, we analyzed discontinuation for each drug separately, avoiding the inclusion of repeated measures in any analysis. In addition, when investigating treatment response for any given antidepressant, we are not able to account for previously taken antidepressants. Due to the nature of our data, we cannot determine if a patient who discontinued treatment was taking any other antidepressants simultaneously. Thus, we cannot account for the potential effects of taking multiple antidepressants or previously taken ones. As an alternative approach to model monotherapy, we conducted our analyses only considering the first prescribed drug for each patient. Although the sample size for this analysis was substantially reduced, which was reflected as lower statistical power, the direction and magnitude of the observed effects is largely consistent with the full results (Additional file 2: Tables S2). Finally, we currently do not have detailed data on treatment regimes such as dosages, which would enable ruling out extremely high or low starting doses as explanations for certain side effects.

In summary, we sought to shed light on which side effects, comorbidities, and demographic factors could influence antidepressant treatment discontinuation. We observed that not all side effects contribute equally to treatment discontinuation. Side effects associated with higher odds of discontinuing treatment included anxiety, agitation, suicidal thoughts, vomiting, and rashes. We showed that participants who also reported comorbid ADHD, PTSD, and a high neuroticism score were at an increased risk for treatment discontinuation. Altogether, our results elucidate which factors could potentially determine antidepressant treatment discontinuation and suggest that discontinuation due to adverse side effects can occur at early and late stages of treatment. We argue that specific drug–factor interactions should be studied in detail to develop new medication recommendations based on demographics and early side effect reports. We suggest that future studies should seek to validate further our results, evaluate discontinuation in monotherapy, estimate the effect of dosages, test for interactions between variables identified here, and investigate the relationship between discontinuation due to side effects, lack of efficacy, and tolerability.

### Supplementary Information

**Additional file 1: Tables S1.** Summary statistics for all associations described in this study.

**Additional file 2: Tables S2.** Summary statistics for associations described in this study only considering the first antidepressant taken as an approach to model monotherapy.

## Data Availability

Summary data on prevalence and effects described in this manuscript are available in the supplementary data. Individual-level data used for this article are restricted due to ethical considerations. Access to the dataset can be granted only after review and approval by the QIMR Berghofer Human and Research Ethics Committee as well as the studies’ principal investigators. Requests to access the datasets should be directed to Nicholas G. Martin at nick.martin@qimrberghofer.edu.au.

## Abbreviations

AGDS: Australian genetics of depression study
SNRI: Serotonin and norepinephrine reuptake inhibitor
SSRI: Selective serotonin reuptake inhibitor
